# Ambient Assisted Living Healthcare Frameworks, Platforms, Standards, and Quality Attributes

**DOI:** 10.3390/s140304312

**Published:** 2014-03-04

**Authors:** Mukhtiar Memon, Stefan Rahr Wagner, Christian Fischer Pedersen, Femina Hassan Aysha Beevi, Finn Overgaard Hansen

**Affiliations:** Department of Engineering, Aarhus University, Aarhus 8200, Denmark; E-Mails: cfp@eng.au.dk (C.F.P.); feay@eng.au.dk (F.H.A.B.); foh@eng.au.dk (F.O.H.)

**Keywords:** Ambient Assisted Living, personal health monitoring, interoperability, healthcare IT, telehealth, electronic health record, participatory development, usability, security and privacy

## Abstract

Ambient Assisted Living (AAL) is an emerging multi-disciplinary field aiming at exploiting information and communication technologies in personal healthcare and telehealth systems for countering the effects of growing elderly population. AAL systems are developed for personalized, adaptive, and anticipatory requirements, necessitating high quality-of-service to achieve interoperability, usability, security, and accuracy. The aim of this paper is to provide a comprehensive review of the AAL field with a focus on healthcare frameworks, platforms, standards, and quality attributes. To achieve this, we conducted a literature survey of state-of-the-art AAL frameworks, systems and platforms to identify the essential aspects of AAL systems and investigate the critical issues from the design, technology, quality-of-service, and user experience perspectives. In addition, we conducted an email-based survey for collecting usage data and current status of contemporary AAL systems. We found that most AAL systems are confined to a limited set of features ignoring many of the essential AAL system aspects. Standards and technologies are used in a limited and isolated manner, while quality attributes are often addressed insufficiently. In conclusion, we found that more inter-organizational collaboration, user-centered studies, increased standardization efforts, and a focus on open systems is needed to achieve more interoperable and synergetic AAL solutions.

## 1. Introduction

Ambient Assisted Living (AAL) systems have a huge potential to meet the personal healthcare challenges and involve citizens in their healthcare through Information and Communication technologies (ICT) [1,2]. The AAL systems provide an ecosystem of medical sensors, computers, wireless networks and software applications for healthcare monitoring. The primary goal of AAL solutions is to extend the time which elderly people can live independently in their preferred environment using ICT technologies for personal healthcare [3,4]. Presently, there is a huge demand for AAL systems, applications and devices for personal health monitoring and telehealth services [5,6]. Moreover, personal health monitoring is setting a trend with increased empowerment of citizens in healthcare, stimulated by the growing awareness and understanding of healthcare concepts and systems, *i.e.*, electronic health medical records, health monitoring systems, and mobile health applications [7]. The AAL systems are also used for telehealth and telemedicine facilities for providing remote healthcare services to the citizens. According to a report by InMedica, telehealth is projected to reach 1.8 million patients worldwide by 2017 for monitoring the post-acute and ambulatory patients [8].

AAL systems consist of medical sensors, wireless sensor and actuator networks (WSANs), computer hardware, computer-networks, software applications, and databases, which are interconnected to exchange data and provide services in an Ambient Assisted environment. Medical Sensors and actuators are connected with the AAL applications and home gateways for sending medical data to the health monitoring systems. The sensors rely on WSANs for connecting with home gateways and healthcare applications [9,10]. The home gateways, also known as smart home gateways, often use a wireless router that provides connectivity to enable multiple applications for real-time health monitoring through home networks [11]. Many of the available sensors used for monitoring blood sugar, blood pressure, and pulse-rate are capable of sending vital signs to the health monitoring systems, so that a caregiver or physician can monitor the patients remotely [12,13]. Moreover, due to increasing availability of portable, wireless medical devices and wide access to data networks the usage of medical devices is continuously growing. According to a recent research report [14], “*Medical devices purchased by consumers used to self-monitor health conditions will account for more than 80% of wireless devices in 2016. The proportion of wireless devices used in managed telehealth programs is predicted to increase from 5% in 2011 to 20% in 2016*”. Another report [15] cites, “*The number of home health monitoring devices in use with embedded cellular connectivity increased from 420,000 in 2010 to about 570,000 in 2011, and is expected to hit 2.47 million in 2016*”. The figures imply that the demand for healthcare devices and ambient assisted living systems is increasing to involve citizens' in personal healthcare, support independent living and economize the healthcare expenses.

In order to produce systems ensuring high-quality-of-service, it is important to consider different aspects of AAL systems to achieve interoperability, usability, security, and accuracy, which are essential requirements of AAL systems. However, the available systems do not consider all aspects of AAL systems having personalized, adaptive, and anticipatory requirements. To identify the essential aspects of AAL systems, we conducted a state-of-the art survey of the literature, which is presented in this paper. We also found other reviews of Ambient Assisted Living systems addressing AAL aspects in general. For example, a recent review by Rashidi *et al.* presents a general technical survey of ambient assisted living platforms, systems, algorithms and standards [16]. A short review by Iliev *et al.* provides a high-level survey of current Ambient Assisted Living systems targeting smart homes, middleware technologies and standards for elderly people [17]. A more distinct review by Antonino *et al.* specifically focuses on architecture-based quality attributes of AAL platforms and evaluates existing frameworks for reliability, security, maintainability, efficiency, and safety properties [18]. In our review, we present the latest research findings and technology advancements of the related AAL systems aspects addressed in these reviews. In addition, we review more aspects of AAL systems, which are also important but not covered in existing reviews.

### Objectives

The objectives of this review are to provide: (1) an overview of the main AAL concepts, (2) a survey of the current state-of-the-art in AAL frameworks, architectures, technologies and standards, and (3) an overview of current usage and real world deployment of specific AAL systems and platforms. Specifically, we want to increase our understanding of how AAL technology has proliferated from being research projects into real world deployment, which has not been covered by previous reviews in the area. Besides, we review more important aspects of AAL systems, including design methodologies, user experience, usability, security, dependability, and accuracy.

## 2. Methods and Materials

This review is primarily based on a state-of-the-art analysis performed through a literature survey of recent AAL frameworks, systems, and platforms. Additionally, we contacted the focal persons and correspondents of various AAL platforms and systems to collect the usage data of the platforms in terms of number of systems in use, supported technologies, standards, deployed devices and applications, service models, and cost per user per system. The analysis and surveys are presented in the subsequent part. We would like to mention here explicitly that this article does not review in depth the applications of ambient intelligence (AmI), robotics, wearable computers, and wireless networks used in AAL, because those are by themselves broad research areas and candidates for separate reviews.

### 2.1. State-of-the-Art Survey and Analysis

**Figure 1.**
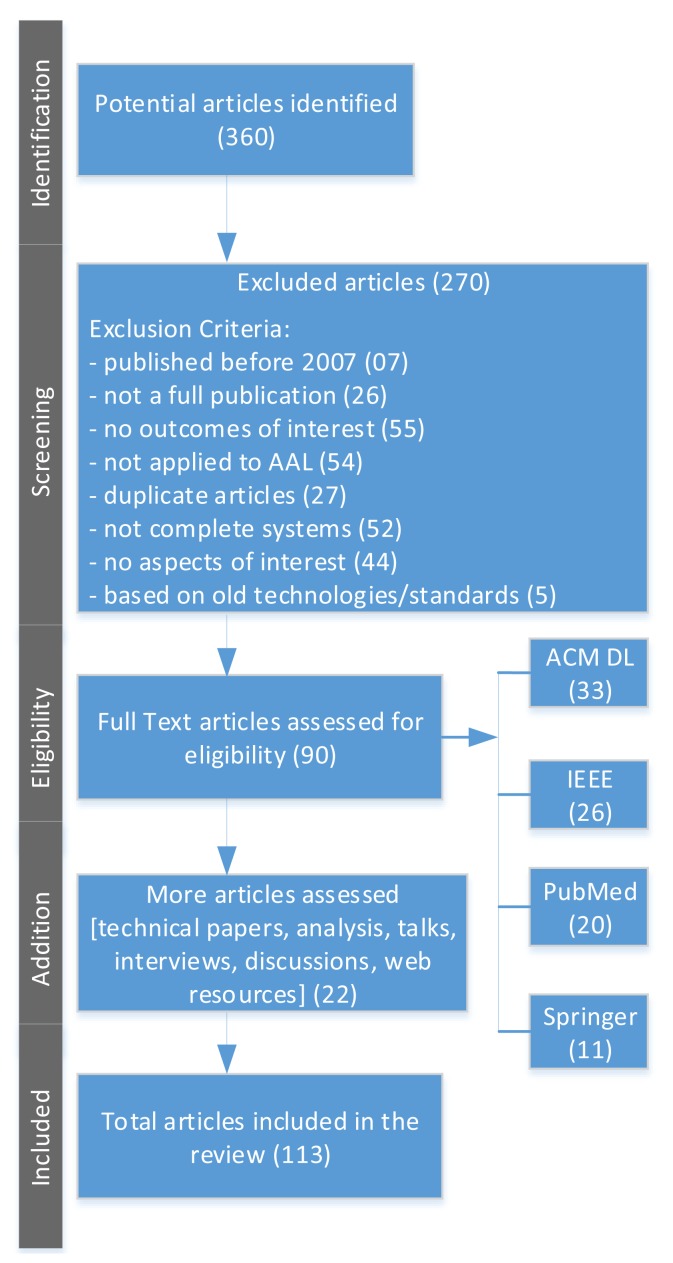
Search strategy and exclusion process for literature review of articles.

We performed a search on the scientific databases ACM DL, IEEE, PubMed, and Springer. We used “Ambient Assisted Living” as the main search criteria and included only those articles in the survey, which address the Ambient Assisted Living frameworks, platforms, and systems. We added the keywords of interoperability, integration, user experience, standards, architectures, security, usability, and design methodologies with the main search criteria. A total of 360 articles were found, from which we selected 90 articles to review from ACM DL (33), IEEE (26), PubMed (20), and Springer (11). A further analysis based on the 90 selected papers uncovered (22) more articles, also including important technical papers, analysis, talks, interviews, technology discussions, and web resources. We selected recent articles from 2007 to 2013 except seven articles published before 2007. The diagram shown in Figure 1 illustrates the review process for this article.

In our analysis, we evaluated contemporary AAL systems, platforms, standards, and technologies to identify essential AAL aspects to be included in the review. Based on the analysis, we categorized different aspects of AAL systems and selected the most important aspects for discussion, which were mentioned in significant number of articles. We classified the articles under the keywords, according to the particular AAL systems aspects addressed in the articles. The corresponding AAL aspects are presented in Sections 3.1–3.8, whereas, Section 3.9 presents the miscellaneous research in AAL. The AAL system aspects identified in our state-of-the-art analysis include medical device interoperability and integration; security, privacy and data protection; design and development methodologies; frameworks and open solutions; quality attributes; technology standards and specifications; and usability and user experience.

### 2.2. Email-Based Survey

We performed an email-based survey to analyze real world usage of contemporary AAL systems and platforms. We wanted to know the number of end-users using certain AAL platforms and systems; technologies and standards used for development, deployment, and integration; supported medical devices; operating systems and programming languages used for development; and cost of the solution per user. For this purpose, we formatted a structured survey form and sent to the correspondents of the AAL systems and platforms identified in the review phase. The survey form is enclosed for reference in Supplementary. We provided a filled form as a guideline to the correspondents for providing data regarding their system or platform. We sent first email requesting data with the survey form, which produced 12 responses. Moreover, eight out of 12 provided data about their solutions. In order to collect more data, we repeated the email as a soft reminder after four weeks and a final reminder email after another two weeks. As a result of this exercise in which we contacted a total of 62 correspondents, we received 40 responses. Out of 40, we received data in 16 responses. The data received through the email survey is discussed in Section 4.

## 3. Results

Table 1 shows the results of the research literature reviewed in our state-of-the-art survey. The first column of the Table 1 lists the essential AAL system aspects identified during the state-of-the-art survey and mentions the number of articles reviewed corresponding to a particular aspect. The second column shows the references of the surveyed literature. More detailed discussion of these aspects is followed by the Table 1 in the subsequent part from Sections 3.1–3.9.

### 3.1. Medical Device Interoperability and Integration

Interoperability and integration of medical devices in healthcare systems processing citizens' vital signs are the most frequently addressed attributes in AAL systems and platforms. Numerous studies have explained its importance and proposed solutions to achieve connected interoperable systems. Recent work by Thelen *et al.* has proposed a telemedicine system for pre-hospital emergency medical services used by the German Emergency Medical Services (EMS) departments [24]. They rely on Health Level 7 (HL7) [90], Integrating the Healthcare Enterprise (IHE) [91] and IEEE11073 [111] standards for data-interchange and device integration. Another AAL system by Hein *et al.* addresses tele-rehabilitation after heart surgery, support to hearing impaired people, and monitoring of daily living activities in a generic Service-oriented Architecture (SOA) using the Open Service Gateway Initiative (OSGi) platform [19]. They implement services to collect the vital signs data from 3-lead Electrocardiography (ECG), blood pressure, and pulse Oximeter sensors. Hanak *et al.* propose a Lifestyle and Health Management System using communication and delivery services in a location-based manner with the Global Positioning System (GPS), Wi-Fi and 3G mobile connectivity [20]. They use Bluetooth-compatible blood pressure and weight measurement devices and wireless physiological sensors for monitoring of body activity, temperature, and stress parameters.

The Continua Alliance deals with Interoperability on a broader level with a focus on IEEE 11073 standard devices [21]. Continua is dedicated for collaborative efforts to improve the quality of personal healthcare and aims to establish an ecosystem of interoperable and connected medical devices for personal healthcare and well-being. The Continua reference architecture uses a Peripheral Area Network Interface (PAN-IF) with medical sensors. Similarly, the Assisted Living Platform (ALIP) in the DALLAS (delivering assisted living lifestyles at scale) program addresses interoperability as the major concern to achieve scalability in medical device communication [22]. The Interoperability Toolkit (ITK) in DALLAS helps integrate systems based on standardizing technologies and interoperability specifications. More research in ALIP i-focus reference architecture solves interoperability issues one step further using the DALLAS Interoperability Layer, which extends the ALIP platform with security features for user and component authentication [23]. To achieve interoperability; Damas *et al.* have used a data-model to transform 11073-compatible device data into a platform-compatible format, so that the home platform should be able to store and process the data without depending upon the device data formats [25]. Their research relies on an OSGi-based system for plug-n-play connectivity of ambient assisted devices. Whitehead *et al.* have elaborated interoperability requirements in a broader spectrum to accomplish a complete accurate electronic medical record system and also ensure health disaster preparedness, emergency handling, clinical support systems and remote health delivery [26,27]. According to them, only open and vendor-neutral platforms deploying systems-of-systems with innovative and intelligent health applications on top of them could realize the vision of “ideal interoperability”. In the same line of thoughts, Galarraga *et al.* investigated the design of robust technical telemonitoring solutions, which are open and interoperable at the same time [28]. Their findings lead to the conclusion that semantic and syntactic interoperability can enable better understanding of telemonitoring systems for lifestyle, genetics, physiology and pathology for management of personal health. AAL systems are used for monitoring a variety of health and well-being parameters also including chronic health problems, as also mentioned by Sanchez-Tato *et al.* in the Health@Home project [29]. Their telecare system monitors cardiovascular patients and ensures reliable data transmission into HL7-compliant data, emergency events detection and management, electronic reminders, and system failure detection. Other studies rely on service-oriented architectures and middleware technologies for integration of scattered business logic and data to provide personal health services ranging from device driver search to accessing Electronic Health Record (EHR) systems. For instance, Pérez *et al.* use the Device Profile for Web Services (DPWS) [92] and other web-services standards, *i.e.*, WS-Discovery, WS-Eventing, and WS-Addressing for integrating ubiquitous devices in the AAL environments [30]. Middleware technologies have a key role for integration and interoperability in AAL systems. Abascal *et al.* have realized the AmbientNet middleware for integration of heterogeneous devices, device discovery, and management of context information [31]. Whereas, the SOA-based research by Corchado *et al.* focuses on the strategical distribution of different sensors and actuators to create the relevant context-awareness capabilities in the AAL systems [32]. In the SYLPH architecture, they have integrated systems using the SOA paradigm over wireless sensor networks and ambient-intelligent applications. Likewise, the SOA-based MPOWER solution by Mikalsen *et al.* have proposed a solution with web-based notifications and alarm services in AAL environments [33].

### 3.2. System Architectures

An AAL solution is an integrated system-of-systems composed of systems, subsystems and components, providing a part of the overall AAL system and its services. The architecture defines the distribution and relationship among the AAL systems, subsystems and components. To exemplify, the conceptual architecture by McNaull *et al.* consists of four architectural layers, *i.e.*, base, data, information, and context layers used for evaluation of the quality attributes of sensors, ambient data, and communication interfaces [34]. The three-layered architecture S^3^OiA by Barbas *et al.* provides a parallel view of architecture for connecting Internet-of-Things (IoT) devices for smart home applications and AAL systems [35]. S^3^OiA integrates objects, people, and applications in an interoperable IoT ecosystem using tripe-space (TS) computing and RESTful web services. They employ TS computing technology for ubiquitous systems to use three dimensions, *i.e.*, subject, predicate, and object for sharing knowledge among heterogeneous devices asynchronously. The open service architecture of the InMyOneWay system by Montes *et al.* detects location of the Alzheimer patients using Global Information System (GIS) system services [36]. Whereas, the architectural solution by Jara *et al.* relies on the ISO/EN 13606 standard to transfer information among distributed medical systems [37]. The proposed architecture ensures home automation, data security, comfort and ambient intelligence to improve quality of life of elderly people. An advanced cloud technology-based architecture by Ekonomou *et al.* uses a Data Capture and Auto Identification Reference (DACAR) platform, which enables controlled access to the clinical services for health monitoring [38]. DACAR is based on Microsoft's HealthVault platform, which provides user interfaces to review health data, define data sharing relationships and manage user profiles [112]. The architecture enforces role-based and service-based authorization using security and privacy policies for integrating homecare applications with hospital systems using a Translation Gateway (TG). The TG translates the access control rights among different integrated systems using trusted and secure connections for a reliable exchange of data. The system architecture can also provide ambient intelligence and runtime verification of safety-critical AAL applications. For instance, the SOA-based architecture by Coronato *et al.* is utilized to develop mechanisms of runtime verification of the correctness properties defined at design-time [39]. The runtime verification techniques are used in the testing phase and during system operation for monitoring of the system behavior.

### 3.3. Security, Privacy and Data Protection

Security and data protection are critical issues in healthcare systems, which exchange and store citizens' medical data. The medical data is security-sensitive. Therefore, legislation provides guidelines to design authorization policies regarding access and usage of the medical data to avoid intentional misuse or accidental disclosure [40,41]. Some of the major threats to data privacy in the AAL system-space are outlined by Rothenpieler *et al.* [42]. Security is considered the central issue for Internet-of-Things (IoT) mobile health devices explored by Doukas *et al.* [43]. They use a Public Key Infrastructure (PKI) based data encryption and digital certificate infrastructure for ensuring confidentiality and integrity of the medical data. The proposed solution collects medical data from mobile/wearable sensors and securely transmits the data to the cloud for access to caregivers and family members of the citizens. More investigations about security system technologies for identification of persons in AAL systems are highlighted by Villacorta *et al.* [44]. An access control model based on Role-based Access Control (RBAC) encompassing organizational models, goals and dependencies is presented by Massacci *et al.* to analyze security and dependability attributes in AAL systems [45]. Security patterns provide guidelines to engineers and developers to benefit from experienced and validated security solutions. In the SERENITY AAL framework, Sanchez-Cid *et al.* have investigated how different roles of software development life cycle, *i.e.*, software engineer, component developer, application developer can use security patterns for modelling and development of an AAL ecosystem [46]. The technical implementation of security- and safety-critical AAL services Venkatesh *et al.* uses knopflerfish-OSGi based architecture [47]. Their solution implements multiple 3DES and AES encryption algorithms for data encryption depending upon required level of security and supported security controls by the devices in AAL systems. Recent research on UniversAAL framework considers security as crucial requirement of AAL scenarios requiring extended access control policies reflecting ambient elements. In this connection, Anton *et al.* propose an authentication and authorization architecture using semantic-based access control for management of distributed identifiers, cross-domain identity federation, multi-device credential management, and context-aware access control [48].

### 3.4. Design and Development Methodologies

Design and development methodologies play a key role in modelling and integration of AAL systems. An analysis by Valderrama *et al.* identifies the major methodologies from the AAL systems design perspective [49]. According to them, service-oriented architectures (SOA), model-driven architecture (MDA), reference model of open distributed processing (RM-ODP), Harmony, rational unified process (RUP), real-time SOA (RT-SOA), and system design through multiple perspectives (S2MP) approaches and methodologies can be used at different levels for development and validation of AAL systems. Likewise, Walderhaug *et al.* add an extra dimension of model-driven development methodology for the development of smart home healthcare applications using domain specific languages [50]. The Model-driven Engineering (MDE) approach is also used to create customized user interfaces at runtime for ambient intelligent systems as discussed by Adam *et al.* and Breiner *et al.* [51,52]. AAL systems are multi-engineering systems consisting of components and equipment from different engineering domains spanning device manufacturing, energy consumption, robotics, computing, and telecommunication. Ras *et al.* have investigated the multi-engineering aspects of AAL systems for the development of a prototype lab ambient intelligence Care and Assistant system (amiCA) [53]. The important multi-engineering aspects of the homecare domain identified by them are low-energy consumption devices, location and context aware devices, medical sensors, computer nodes, robotics, *ad hoc* communication networks, adaptive learning systems, virtual acoustics and audio engineering, interactive systems, ambient intelligence and usability. The user-centered and participatory development methodology with fast prototyping, involving interactive feedback from end-users produces better usable systems, as also argued by Kieffer *et al.* [54]. In their Keep-in-Touch project, they have used the participatory development paradigm to achieve multimodality, accessibility, adaptability, and usability in AAL systems. Another user-centered approach by Aquilano *et al.* proposes socio-medical services involving caregivers, elderly subjects, judicial researchers, and technological experts for the development of better usable AAL systems [55]. For their prototype, they have used different modules including health, wearable, sensor network, and ambient-intelligence as essential components of a complete and usable AAL system. O'Grady *et al.* on the other hand, discuss the evolutionary development of AAL systems outlining major technical and methodological approaches used for the development of AAL system components [56]. They apply the evolutionary development for tracking the elderly people in different parts of the home using infrared sensors as collaborating agents. Other methodologies are built upon formal validation and reasoning. For instance, Parente *et al.* rely on the formal modelling tools and techniques for Fault Tree Analysis (for dependability analysis), Evidential Reasoning (for impact of evidence analysis), Temporal Logic (for temporal reasoning), and Markovs Model (for system states) for ambient intelligence and analysis of system properties [57]. Semantics of ambient contents in AAL systems are investigated by Lugmayer *et al.* to add intelligence through interpretation algorithms and techniques and also collect attributes of context awareness and mobility of ambient data [58]. The NECESITY project by Botia *et al.* also defines a complete system development process for high-level modelling, formal analysis, architectural design and evaluation of in-home monitoring system for healthy independent elders [59].

### 3.5. Conceptual Frameworks and Open Solutions

System modelling and implementation of AAL systems is mostly led by the conceptual frameworks, architectures, and open solutions. There is substantial research in this direction; however, we will mention only some of the major frameworks and architectures for brevity. Tazari *et al.* present a reference model for AAL as the UniversAAL platform for large-scale integration of different AAL systems and solutions [60]. Their objective is to build a consensus among the AAL community and consolidate their efforts to produce technically feasible and economically affordable standardized AAL systems. The main conceptual components of the proposed domain-specific models in the UniversAAL platform consists of AAL services, network artefacts, AAL spaces, and AAL platforms, which lead the development of AAL systems. Schmidt *et al.* believe that AAL systems are too diverse and cannot be provided as commercial-of-the-shelf (COTS) solutions [61,62]. To cope with the diversity challenges of AAL systems, they propose an open middleware OpenAAL to enable easy implementation and configuration through situation-dependent and context-aware personalized AAL services. The middleware platform is built on the OSGi architecture and Business Process Execution Language (BPEL) used for deployment of loosely-coupled platform services and ontologies-based information to capture sensor and situation inputs in ambient environment. OpenALL was preceded by the SOPRANO project with similar objectives to support independent living and social participation empowering AAL systems with sensors, actuators, smart interfaces and artificial intelligent [63]. In the automatic smart home framework of the U-Health project, Agoulmine *et al.* have proposed four main conceptual layers, *i.e.*, sensors & actuators, home communication network (HCN), automatic decision-making system (ADMS) and safety & healthcare services, as the most important elements of smart homes used for personal healthcare monitoring [64]. Also, the AmiVital interaction framework architecture by Jiménez *et al.* exhibits a relationship among functional and technological services [65]. Besides, it also provides components for context management, knowledge management and device connectivity. The architectural layers of the Hydra middleware by Eisenhauer *et al.* define the conceptual levels of ambient-intelligence systems as physical (Zigbee, Bluetooth, WLAN), OS (Windows, Linux, TinyOS), Hydra middleware (for applications and devices) and application (workflow, user interfaces, configuration and business logic) [66]. Their middleware provides security to the applications and devices, which are connecting through Hydra. Similarly, the OpenCare framework by Wagner *et al.* extends the conceptual architecture in four logical tiers of home, mobile, central, and public to provide a complete pervasive and connected infrastructure for healthcare monitoring [67]. The OpenCare framework is implemented as the Sekoia platform used for personal healthcare monitoring through locally-installed healthcare applications and telehealth services [68].

### 3.6. Quality Attributes

Quality attributes have a greater impact on the usability of AAL systems. Some of the essential quality attributes in AAL platforms are presented by Oliveira *et al.* They have evaluated different AAL projects including Alhambra, OpenAAL, UniversAAL, PERSONA, and Hydra [18] for quality attributes. In particular, their evaluation is based upon the quality attributes of reliability (recoverability), security (encryption and access control), maintainability (changeability and installability), efficiency (interoperability, resource consumption) and safety (single-point-of-failure, usage patterns). Accuracy is regarded as an important quality attribute for integration of data-intensive AAL systems. Jara *et al.* realize the importance of accurate health monitoring with the examples of glucose-level data [69]. According to them, glucose values are affected by many factors such as illness, psychological stress, drugs, fluids, and meal plans. Moreover, it is essential that diabetic patients using automatic insulin infusion should get an accurate dosage of insulin. They propose a diabetes therapy management system for AAL environment. Their home gateway is capable of identifying the patients through RFID and use the insulin infusion protocol based on artificial intelligence for adaptive insulin infusion through self-monitoring blood glucose sensors. The quality of data, information and contextual knowledge is crucial in AAL systems according to McNaull *et al.* [34]. They propose four layers of the AAL system architecture including base, data, information and context layers to investigate the quality attributes of sensors, ambient data, and communication interfaces. Accuracy in self-measurement of the vital signs is also a critical requirement. Wagner *et al.* have investigated the accuracy of self-measurement of blood pressure (BP) data, when patients are required to self-measure their BP at a regular basis in an unsupervised setting [70]. Their research uses a context-aware system, ValidAid, to gather context information of BP self-measurements to investigate the patients' adherence to the measurement recommendations. They conclude that understanding the measurement context, including time-rested before measurement, posture, noise-levels, and several additional parameters, is crucial for obtaining valid data from BP measurements and other types of self-measurements and self-care activities in the unsupervised setting. Finally, they conclude that current state-of-the-art telemedicine and AAL equipment cannot guarantee a sufficient data-quality, leading to serious misdiagnosis and potential treatment errors.

Usability is considered as one of the major quality attributes of AAL systems, mainly because the end-users have no technical expertise in handling different devices, applications, network equipment, gateways, and other infrastructural components. Moreover, the end-users also include patients, elderly people, and people with disabilities or specific health deficiencies. Considering these aspects, Kim *et al.* provide an extensible architecture for seamless integration of heterogeneous protocols and vendor-specific devices in a secure and interoperable manner [71]. The seamless integration of devices and protocols provides usability as end-users are not involved in handling device drivers and applications. The solution provides automatic connectivity and configurations for ready-to-use applications and devices. Their implementation is based on the OSGi platform and cloud technologies. OSGi is used at the home gateway for plug-n-play deployment and installation of heterogeneous devices during runtime. The home gateway connects to the cloud to download device drivers and applications from application store. Usability of medical devices, applications and systems is also elaborated by Boulos *et al.* [72,73]. To ensure maximum usability at different levels in the eCAALYX solution, they provide docking stations (for charging mobile devices), large-touch screens, automatic and seamless system updating, limited user interface screens and avoiding error prompts. Kleinberger *et al.* believe that accessibility, usability, and learning are key features of AAL systems. They emphasize that the disabled and elderly should be trained to use future technology interfaces, so that they might be capable of using personal healthcare devices without technical support [74]. Beyond the primitive services, they outline adaptivity, anticipatory human-computer interaction, heterogeneity, integration and, domain knowledge formalization as the key challenges for AAL success and acceptability. Likewise, Jiménez *et al.* propose a user interface framework based on adaptive interactions to enhance the usability of AAL systems [65]. The proposed middleware adapts user interfaces based on the user preferences and contextual data retrieved from the sensors. The challenges faced by usability engineers in designing user interfaces of multi-device and cross-platform AAL systems are discussed by Mourouzis *et al.* [75]. They propose a user interface adaptation platform infrastructure using a decision making specification language (DMSL) server and easy-to-use adaptive widget library. The AAL systems require personalized configurations; which implies that the medical sensors, network, system and applications are procured, installed, and configured corresponding to the requirements and preferences of an individual or a group of users. Installation and configuration of devices and applications is complex and requires intervention from technical personnel to set the environment for users as also agreed by Marinc *et al.* [76]. They propose an interactive and architectural configuration approach for comprehensive AAL environments targeting different types of users based on the levels of technical expertise as expert, normal and impaired users. Similarly, López-de-Ipiña *et al.* believe that middleware platforms supporting automatic integration of sensors and services in AAL-enabled homes can increase interoperability, integration and usability [77]. The infrastructure for the ZAINGUNE platform deployed by them consists of an OSGi-based middleware supported by rule-based reasoning engine, sensors, IP surveillance camera, VoIP telephony, and messaging actuation mechanisms. Automatic user identification through RFID tags by Iglesias *et al.* evaluates the usability of an AAL system for health monitoring using wirelessly connected medical sensors [78]. They conclude that automatic user and device identification reduces the complexity and increases the user acceptability. In a similar way, Busch *et al.* performed experiments to achieve unobtrusive, non-stigmatizing, and continuous acquisition of vital signs from medical devices into the computer-based systems to enhance AAL systems usability [79].

The ambient data in AAL systems deals with the healthcare of citizens. Any errors in the data created by sensors, computers processes, or network transmission can have a profound effect on the real-time operation of AAL systems. The consequences due to errors can be unfavorable to citizen's health. Realizing the importance of data quality in AAL systems, McNaull *et al.* have analyzed the data quality issues in medical sensors, activity sensors, location sensors, wireless communication sensors, and context-sensors used in AAL environments [34].

Dependability is one of the important quality parameters in AAL systems as mentioned by Rodrigues *et al.* According to them, the poor quality of system availability, safety, and integrity can lead to emergency and fatal consequences for lonely elderly citizens relying on home-based personal healthcare systems [80]. Their research technique uses the PRISM model-checking tool to validate UML behavioral models and analyze dependability and sensitivity properties of AAL system models. Moreover, AAL systems also require runtime verification of data and business processes to achieve reliability in safety-critical scenarios. Reliability of safety-critical ambient intelligence applications is evaluated by Coronato *et al.* for continuous monitoring of systems-in-execution, against correctness properties [39,81]. The approach uses rapid prototyping to verify several system characteristics using structural and behavioral patterns for runtime verification targeting location of the mobile users within smart homes.

### 3.7. Technology Standards and Specifications

Technology standards and specifications provide the basis to achieve interoperability, integration, and scalability through standardized protocols and data models. An end-to-end standard-based patient monitoring solution by Martinez *et al.* transforms the medical data from the X73 Point of Care Medical Device Communication (PoC-MDC) [82] devices into the EN13606 standard and stores at an electronic healthcare record (EHR) server [83,84]. EN13606 is the extensible European standard for interoperable EHR, which allows adding new medical information to the system, using archetype model that gathers domain concepts for extension [85]. Another work by them discusses how X73-PHD (personal health devices) can be used in the intensive care, hospital admission, and blood donor centers to check vital signs including blood pressure, heart-rate, temperature, and breathing-rate using X73 monitor and manager applications [111]. The X73 monitor converts the data from proprietary formats into X73 using a conversion tool. The ASTM F2761 standard considers the safety requirements in medical devices and systems as the main agenda [86]. It defines a framework for integrated clinical environment focusing on “device interoperability towards safety” for medical devices and equipment. The ASTM F2761 defines requirements and procedures for design, verification and validation of integrated clinical environments incorporating model-based integration of systems. Similarly, the differences in medical device communication standards for ECG are investigated by Trigo *et al.* [87]. They have proposed an x73-PHD model for an ECG device and evaluated other widely used ECG standards including, Standard Communication Protocol for Computer-assisted Electrocardiography (SCP-ECG), HL7 Annotated Electrocardiogram (aECG), Medical Waveform Format Encoding Rules (MFER), and Digital Imaging and Communications in Medicine (DICOM). Whereas the research by Ferreira *et al.* focuses on web-service based AAL environment to collect vital signs from medical sensors and transform the data in the Continuity of Care Record (CCR)—ASTM E2369 standard [88]. The CCR provides snapshot data to patients' clinical, demographic, and administrative data in a structured electronic format [89]. CCR is used to develop clinical information systems to integrate CCR data as a part of HL7-Clinical Document Architecture (CDA) document, which enhances system scalability. Further standards used in AAL systems are related to wireless sensor networks (WSNs) as most of the medical devices are connected wirelessly with home gateways and homecare systems. In a review, Zubiete *et al.* have analyzed different wireless sensor technologies (*i.e.*, ZigBee, Bluetooth, RFID, IEEE 802.15.4) used for health monitoring applications including fall detection, ECG, diabetes, scoliosis, body fluid, and breath-monitoring [93]. Likewise, the Common Accessibility Profile (CAP) in the ISO/IEC 24756 standard checks the accessibility options for users. Using this standard, Sala *et al.* have proposed a method and an Integrated Development Environment (IDE) to verify the accessibility constraints of an AAL system configuration against user capabilities [94]. The authoring and simulation environments in the proposed human centered design (HCD) framework allow designers to create, deploy components interactively and simulate the AAL solutions. Similarly, a smart home solution by Caballero *et al.* offers services in an ambient environment for electrical power management and health monitoring devices using the ZigBee Cluster Library (ZCL) to achieve interoperability and affordable AAL services [95].

### 3.8. User Experience

Several research studies have investigated the user experience of AAL systems in nursing homes and private homes. An assistive technology framework by Panek, *et al.* shares the experience of several living labs established at nursing homes in Austria [96]. The living labs at Schwechat use ZigBee-based sensors for temperature, Reed Relay-control, and acceleration measurements. A related exploratory study by Beringer *et al.* is based on case studies investigating acceptability of AAL devices and systems among the elderly users [97]. The findings suggest that there is a high interest and impact of AAL systems among the elderly users. However, the users are concerned about independence, security, privacy, and behavioral freedom within the home-space under the watchful eye of an AAL system. This is also verified by Ziefle *et al.* They found similar user attitudes in video-based monitoring for long-term care of elderly and disabled people in the smart homes [98]. In a user experience study, Ibarz *et al.* performed tests to validate user acceptability, usability, feasibility, and long-term sustainability in their exploratory research studies targeting elderly people with disabilities spanning five European countries [99]. Sun *et al.* believe that socialization can keep the AAL users connected with each other for better quality of life [100]. They suggest virtual communities to enhance the user experience for social intelligence in AAL systems supported by virtual reality applications. Some of the research studies address the emergency situation handling using AAL technologies. Kleinberger *et al.* performed experiments to measure the accuracy in monitoring of emergency situations using personal emergency response systems (PERS) and automatic behavior monitoring systems (ABMS) techniques [101]. They found positive results for long-term and short-term monitoring of elderly people in smart homes, equipped with appropriately positioned sensors at different locations.

### 3.9. Miscellaneous Research in AAL

In this subsection, we present the miscellaneous research in healthcare systems which is closely related to, but not discussed under the specific aspects of AAL presented in the previous subsections (3.1 to 3.8). In the broader scope of healthcare systems research, Blobel *et al.* have presented interaction and integration paths in eHealth discussing the standards, network protocols, applications, and middleware technologies [102]. They have defined a comprehensive set of non-functional requirements and services in mobile personal health including data security, usability, transparency, and scalability. Whereas, the reviews by Demiris *et al.* and Hensel *et al.* have categorized different smart home technologies and applications as physiological, functional, safety, security, social interaction monitoring, and cognitive & sensory assistance [103,104]. They ensue that earlier solutions focused more on intensive monitoring of the patients, whereas the recent systems are inclined towards empowerment of citizens through increased interaction and awareness among users and systems. They also found that current AAL systems research lack in user-perception, usability, ethical frameworks, informed consent mechanisms, and technical system evaluation procedures. Besides, security, privacy, reliability, and robustness are perceived by them as the main challenges in AAL systems. AAL system design involves multi-organizational interactions as different AAL components including medical sensors, device drivers, medical applications, medical records, and enterprise infrastructures, which are provided and managed by different organizations. The importance of organizational cooperation for AAL systems is elaborated in the VirtualECare project by Novais *et al.* [105]. The VirtualECare architecture service-point-of-view comprises of organizations, recommendations, technical infrastructure, and identity management as the main components for health monitoring. Dohr *et al.* have connected smart objects with closed-loop healthcare services to setup an infrastructure for AAL scenarios [106]. The key parameters identified by them for achieving “IoT for Ambient-assisted Living” are identification, location, sensing, and connectivity to achieve secure, safe, and social independent living. Similarly, the AAL solution by Takács, *et al.* provides integrated functionality for mental monitoring, mood assessment, and physical & relaxation exercises beyond basic health management services [107]. Their Ambient Facial Integration (AFI) technique uses photo-realistic animated faces for identifying emotional facial expressions (happiness, surprise, fear, sadness, and anger), non-verbal feedback, and body language for elderly persons. The AFI techniques are also used by them to identify the state of RFID-tagged fruits to check if those are fresh or stale or edible out of their physical properties, *i.e.*, color, skin pattern. The concept of “ambience” is not limited to the home of elderly persons. Huertas *et al.* believe that it is equally important that the elderly people should be provided with health and information services while they are outside in the surroundings of their homes. Their concept of the Information and Assistance Bubble aims to achieve this objective [108]. The Bubble is defined as the physical space, which extends the mobility of the elderly users and is accessible within a wireless network providing health and information services. Montague elaborates a commercial Vitalsense responsive healthcare platform for connected personal healthcare to monitor ECG, heart-rate, respiration-rate, and accelerometer devices [109]. The Vitalsense platform covers a wide range of sensors and monitoring applications.

This concludes the section discussing the results of our state-of-the-art survey of AAL frameworks, platforms and systems, which is the primary focus of this review. In this section, we categorized the essential aspects of AAL systems and discussed the latest research in the corresponding areas. The next section supplements our state-of-the-art literature review with current status and usage data of contemporary AAL frameworks, systems and platforms, collected by us through an email-based survey as discussed in Section 2.

## 4. Usage of Contemporary AAL Systems and Platforms

We conducted an emailed-based survey to analyze the real world usage of contemporary AAL systems and platforms as detailed in Section 2.2. Based on the survey, Table 2 shows the data received from the correspondents and focal persons of different AAL system, platforms or solutions. A total of 62 correspondents were contacted, of which 40 responded. As shown in Table 2, 16 of the 40 correspondents communicated the required information about users, technologies, supported devices, platforms, standards, and cost. Whereas, 24 correspondents did not send any data due to project or business confidentiality policies. Of the 16 positive responses, 12 reported actual information concerning the number of end-users, staff users, and systems deployed, while 6 provided the cost per system per user. Most provided the information about technology, standards, supported devices, and platform. From the usage point-of-view, OpenCare/Sekoia [68], TemRas [24], WayFIS [113], and REMOTE [75] reported the maximum number of end users 2200, 300, 200, and 166, respectively (see Table 2). The medical devices supported by different platforms were mainly blood pressure, glucometer, pulse oximeter, fall detector, and weight scale. Besides, other devices such as heart-rate monitor, dehydration sensor, subcutaneous pump, intraoral device, and electronic stethoscope were also reported being used. The standards and specifications in-practice are dominated by the IEEE-11073 standard [21] for device communication and HL7 [90] for healthcare interoperability. Also, ISO standards are used for different purposes including ISO/IEC 29341 (UPnP Device Architecture), ISO-13485 (Medical devices—Quality management systems), and IEC 60601 (Requirements for medical electrical equipment and medical electrical systems used in the home healthcare environment). The operating systems being used are Windows, Linux, Android, and Symbion. Java, C#, C++, PHP, and Python are the major programming languages used for development of AAL platforms and applications. The communication among distributed architectures is achieved through the HTTP, SOAP, and RESTful protocols. The network architecture consists of WiFi, Ethernet, as well as ZigBee and Bluetooth for personal area network. The cost of the platforms ranges from EUR 500 to EUR 20,000 depending upon what is included in the platform and services.

## 5. Discussion

We found that a significant number of research and industry organizations are active within the AAL field. These organizations have engaged in a high number of recent research and development activities addressing different aspects of AAL, and publishing their results for the community as presented in Sections 3.1 to 3.9. However, as compared to the extensive research efforts presented, the real world usage appears to be fairly limited and confined to a few devices and standards being applied beyond the pilot-study level. An explanation for this could be the wide gap among the requirements of real world AAL system scenarios and the capabilities of currently available solutions and enabling technologies. Moving from research prototypes and pilot studies is difficult and requires more technical, economical, and organizational resources and commitment to succeed. Also, there are large differences in user populations leading to more complex user experience and socio-technical requirements, while the requirements to standardization and certification incurs higher efforts and cost of executing the research and development organizations, as compared to traditional system development.

Through our state-of-the-art survey, we learnt that research in AAL systems is getting aligned strategically with real world scenarios as more requirements are being identified with user-centered and participatory research methodologies. Besides, the ICT advancements in hardware and telecommunication systems, software engineering tools, techniques, and model-driven approaches are supporting the development of AAL systems to a greater extent in the technologically-heterogeneous service-oriented architectures. The formal modeling and validation tools are enhancing the reliability and dependability properties. These are not yet gaining ground in real world scenarios. Significant results have been achieved in terms of enhanced interoperability and integration among medical sensors and healthcare applications used in AAL systems, through the emergence of standards for data and device integration, as well as open frameworks for providing access to this. These include the IEEE 11073, HL7, ISO-13485, and IEC 60601 standards for exchanging data, while standards such as ZigBee, ZWave, Bluetooth provide wireless communication interfaces between the devices, and solutions. However, despite huge claims by device and application vendors, the system engineers are still dealing with closed solutions and proprietary protocols for exchanging the very basic medical data among devices and applications. Also, sharing specialized software components and applications between different projects is not possible without rewriting significant portions of the code, hampering the proliferation effort of relevant AAL applications and components even more.

Even so, the ‘Healthcare IT’ industry has promptly entered into the scene, but has failed to provide interoperable and affordable systems to citizens and support caregiver and governmental organizations. Opening up for standardized device data exchange, as mandated by the Continua Alliance, does not imply that systems become more open for sharing applications and algorithms. Opposed to this, the end-users are mainly concerned about the security and privacy of their personal medical data and want to be assured that their personal data is not exposed to unauthorized ends and that they can combine the AAL services from different vendors that they need. This is in contrast to many vendors and data providers relying on cloud and other server-based services for uploading and storing personal health data and other information, and not allowing other AAL systems access to accumulated data. The freedom of the individual patients for choosing the data-handling strategy has yet to be addressed by the AAL vendors, especially industry, as well as the freedom of choosing the AAL applications and services they want to use, rather than being handled as a silo package.

The conceptual frameworks, platforms and architectures provide guidelines for the software architects and developers to understand the requirements of AAL systems. However, only few of those have produced sustainable systems. Most frameworks focus only on a few aspects, ignoring the requirements of an entire AAL system as seen from different stakeholder and design perspectives. As a result most of the reviewed frameworks produce partial solutions only that cannot support full-fledged solutions ready for real world deployment. These findings are further supported by the usage-data collected during our survey. Here, we found that only 16 organizations were willing or able to provide relevant feedback out of the 62 projects included in the email survey. Almost 32% of the potential respondents, 20 out of 62 did not even respond to the email survey, despite repeated communications to different contact persons of the individual project and organization. Out of the 40, respondents, a majority did not feel their contribution would be relevant or did not know what the project status was, while another faction referred to “business confidentiality policies” as the reason for not sharing their data. Thus, we can only speculate on the real motives of the non-respondents, but we assume that the 16 full-respondents represent the AAL projects that are ongoing, while the others most likely represent the AAL projects that were closed after end-of-funding. Furthermore, out of the 16 respondents, only 12 provided usage data, while six provided actual system costs. Obviously, the AAL community would have benefitted significantly more from a higher response rate.

It is also evident from the survey that AAL research is not sufficiently focused on solving the most critical problems identified in this and other previous reviews, including interoperability, usability, security, reliability, and the quality of the user experience. Instead, the research most often only deals with the isolated aspects of AAL. Moreover, there is a disproportional high overlap between the reviewed AAL studies research objectives and activities, which results in a lack of innovation and focus for achieving usable and affordable AAL systems. The overall vision of producing heterogeneous, open, and reusable infrastructure platforms and components, consisting of diverse software and hardware elements spanning from reasoning algorithms to specific healthcare and assisted living devices, is still to be presented in the literature. The studies that do report on such open and heterogeneous systems do not sufficiently exemplify heterogeneity with specific inter-project and inter-vendor examples, but is usually focusing on presenting a few devices at the intra-project level with in sufficient details.

If the AAL community is to achieve the desired significant gains and reach the objectives set forth in the common AAL vision, the AAL organizations must open up for increased collaboration and integrate more systems into their evaluation sites. This calls for more open standards and open source solutions. Eventually, this could lead to more AAL services and devices being deployed at the existing end-user population, and more evaluation data reaching the AAL community, and thus provide the synergetic effects that are needed for the AAL vision to prevail.

## 6. Conclusions

The usage of ambient assisted living platforms and systems is growing with time as strategists and policy-makers have drawn the attention of governments, scientists and IT industry to the alarming numbers of growing elderly population. However, as we have shown in this review, current AAL solutions do not consider all the essential aspects of AAL systems and lacks the required interoperability and integration properties. Besides, usability, reliability, data accuracy, cost, security, and privacy are the major challenges for current AAL systems, resulting in unacceptable installation and deployment complexity, unappealing user interfaces, security threats, lack of quality-of-user-experience, and higher costs. Moreover, as the review has shown the end-users continue to require significant technical support and supervision from skilled IT personnel for keeping the home based AAL systems operational for daily use. This does not only hinder the objectives of supporting independent-living but also makes the solutions more expensive for the elderly population and their sponsors to acquire and maintain, as well as delays the proliferation into both public and private care institutions, and private homes.

Furthermore, we found that more validation and user experience studies are required to produce better AAL systems with additional user feedback and participatory development approaches. Design methodologies and paradigms can play a key role for evolving more robust, consistent, and reliable AAL solutions. Inter-organizational collaboration can also enhance the quality, provide outreach to end-users, and normalize the cost of AAL systems. For that, it is important to involve citizens, caregivers, healthcare IT industry, researchers, and governmental organizations in the development cycle of AAL systems, so that end-users can benefit more from the collaborative efforts.

This review has identified a high number of research and industry organizations who are currently active within the AAL field. However, the extensive research effort has not yet led to a significant proliferation of technologies into real world usage. Thus, our review only uncovered 12 projects which had continued their projects beyond the pilot phase and deployed their solutions into the real world, either at care facilities or private homes. Also, the review found, that despite the wide spread aims of supporting for openness and heterogeneity, most projects have not yet addressed this sufficiently for allowing for any substantial reuse of software and hardware components to become a reality. Thus, we argue that one of the major challenges facing the AAL field in the coming years is to reach the goal of openness for achieving more interoperable and synergetic AAL solutions that can gather the critical mass needed for succeeding with our efforts as a field. This challenge could arguably be conceived as the largest barrier to overcome.

## Figures and Tables

**Table 1.** Main aspects of AAL systems and surveyed literature.

**AAL Aspects (No. of articles reviewed in state-of-the-art survey)**	**Surveyed Literature (References of the surveyed literature)**
Medical Device Interoperability and Integration (15)	[19–33]
AAL System Architectures (6)	[34–39]
Security, Privacy and Data Protection (9)	[40–48]
Design and Development Methodologies for AAL systems and Services (11)	[49–59]
Frameworks and Open Solutions (9)	[60–68]
Quality Attributes -Usability, Accuracy, Dependability, Availability, Reliability (17)	[18,34,39,65,69–81]
Technology Standards and specifications (14)	[82–95]
User Experience (06)	[96–101]
Miscellaneous Research in AAL Systems (08)	[102–109]
Reviews/Surveys in AAL (7)	[1,5,16–18,93,110]

**Table 2.** Data showing usage of contemporary AAL platform and systems.

**No.**	**Project Title/Description/URL**	**Systems Deployed (#)**	**End Users (#)**	**Staff Users (#)**	**Homes Deployed (#)**	**Medical Devices Supported**	**Standards**	**Operating systems**	**Programming Languages**	**Service Model**	**Cost/system/user (EURO)**
1	**AALuis**—Ambient Assisted Living user interfaces, http://www.aaluis.eu/	N/A	N/A	N/A	N/A	N/A	ISO/IEC, 29341-x (UPnP Device Architecture)	Windows, Linux, Mac OS X, Android	Java, ECMA Script, XSLT	N/A	N/A

2	**AMICA**—Autonomy, Motivation & Individual Self-Management for COPD patients, http://www.aal-europe.eu/projects/amica/	30	30	2	10	N/A	HL7, X.509, Bluetooth LE/Continua, ISO 13485, ISO 60601-1-4, ISO 62304, ISO 9241	Windows XP/Vista/7/8, Android	C#, C++, Java, WSDL	SOAP, .NET	990/Year

3	**AAL-ALFA:** Active Living for Alzheimer-patients, http://www.aal-alfa.eu/	N/A	N/A	N/A	N/A	N/A	N/A	Android 4.1	Java	N/A	N/A

4	**HELP:** Home-based Empowered Living for Parkinson's disease Patients	24	24	9	24	Blood pressure, Parkinson sensor, Subcutaneous pump, Intraoral device	Zigbee Health Profiles, IEEE 11073	Windows Mobile, Android	C++, Java	OSGi	3500

5	**GoldUI:** Adaptive embedded human interfaces designed for older people, http://www.goldui.eu	N/A	40	20	N/A	N/A	ISO/IEC 40500, ISO/IEC 18036, ISO 9241-151	Windows XP/Vista/7/8, Linux, Android	PHP, Java, JavaScript,Postgre SQL	SaaS	N/A

6	**WayFiS:** Way Finding for Seniors	N/A	200	50	N/A	N/A	ISO 20282, ISO/IEC 27002, ISO 17267:2009	Windows XP/Vista/7, Linux, Android	PHP, Java, JavaScript, Postgre SQL	SaaS	N/A

7	**MyGuardian:** A Pervasive Guardian for Elderly with Mild Cognitive Impairments	N/A	N/A	N/A	N/A	N/A	ISO 6709:20, ISO 20282, ISO/IEC 27002	Windows XP/Vista/7, Linux, Android	PHP, Java, Postgre SQL, .NET	SaaS	N/A

8	**HOMER**—Home Event Recognition System	50	50	20	50	Continua Health certified devices	ISO/IEEE 11073-10471	Windows XP/Vista/7/8, Linux	Java	OSGi RESTWeb Socket, JSON	N/A

9	**HOPE**—smart home for elderly people	7	37	10	6	Pulse counter, Fall detector, Panic-Button	HL7	Windows XP/Vista/7/8	C++, VB.net, Java, C#	N/A	N/A

10	**NACODEAL:** NAtural COmmunication DEvice For Assisted Living, http://www.nacodeal.eu/en/	N/A	N/A	N/A	N/A	N/A	N/A	Windows XP/Vista/7/, Linux	N/A	REST	1000

11	**PAMAP**—Physical Activity Monitoring for Ageing People, www.pamap.org	3	30	5+	30	Heart rate monitor	HL7	Windows, Linux	C++, Java	SOAP	N/A

12	**REMOTE** (Remote health and social care for independent living of isolated elderly with chronic conditions), http://www.remote-project.eu/	17	166	73	20	Zephyr bio harness, Dehydration sensor, Blood pressure device, weight scale	HTTP, SOAP	SymbionOS	Java, LWUI, JADE	SOAP	30–35

13	**SmartTouch:** Interaction as simple as touch, http://ttuki.vtt.fi/smarttouch/www/?info=intro	1	0	5	0	Weight-scale, Glucose Meter, Blood Pressure	N/A	Windows XP	C#	SOAP, .NET	N/A

14	**SOFTCARE**, http://www.softcare-project.eu/overall.php	4	4	2	4	N/A	N/A	Windows XP/Vista/7/8	Java, C	SOAP	500

15	**TemRas:** Telemedizinisches Rettungsassistenzsystem, Telemedical Rescue Assistance System	6	300	13	0	Monitor/Defibrillator for Emergency Medical Services (Philips HeartStart MRx), Electronic Stethoscope (3M-Littmann E3200)	HL7	Windows 7/2008 Server, GNU/Linux	Java, Python, C#	AMQP, FTP	20,000

16	**OpenCare Platform @ Sekoia** http://opencareproject.wikispaces.com/home	2000	2000	200	2000	Blood Pressure, Glucometer, Pulse-Oximeter, Weight-scale and other Continua certified devices.	HL7	Windows	C#	SOAP, REST	N/A
